# Correction: Clay minerals evidences for cold-warm fluctuations in the early Silurian

**DOI:** 10.1371/journal.pone.0350353

**Published:** 2026-05-28

**Authors:** The PLOS One Staff

## Notice of Republication

This article was republished on March 24, 2026, to rectify an error in the title introduced during the typesetting process. The publisher apologizes for this error. Please download this article again to view the correct version. The originally published, uncorrected article and the republished, corrected articles are provided here for reference.

## Supporting information

S1 File Originally published, uncorrected article. (PDF)

S2 File Republished, corrected article. (PDF)
