# Supplementary material for: Correction: Clay minerals evidences for cold-warm fluctuations in the early Silurian
Source: PLoS One. 2026 May 28;21(5):e0350353. doi: 10.1371/journal.pone.0350353 (PMC13218466; doi:10.1371/journal.pone.0350353)
Supplement: S1 File — (PDF) [file pone.0350353.s001.pdf]

PLOS One

RESEARCH ARTICLE

Clay minerals evidences for cold-warm fluctuations in the Early Silurian

Zhibo Zhang[1]*, **Jiaming Zhang**[2]*, **Hengye Wei**[3], **Huan Li**[4,5], **Yinghai Guo**[2], **Chunlin Zeng**[6]

1 College of Resources and Environment, Yili Normal University, Yining, Xinjiang, China, **2** School of Resources and Geosciences, China University of Mining and Technology, Xuzhou, China, **3** School of Earth Science and Technology, Southwest Petroleum University, Chengdu, China, **4** State Key Laboratory of Critical Mineral Research and Exploration, Central South University, Changsha, China, **5** School of Geosciences and Info-Physics, Central South University, Changsha, China, **6** National and Local Joint Engineering Research Center of Shale Gas Exploration and Development, Chongqing, China

* zzb397132190@126.com (ZZ); zjming@cumt.edu.cn (ZJ)

Check for updates

OPEN ACCESS

Citation: Zhang Z, Zhang J, Wei H, Li H, Guo Y, Zeng C (2025) Clay minerals evidences for cold-warm fluctuations in the Early Silurian. PLoS One 20(10): e0335236. https://doi.org/10.1371/journal.pone.0335236

Editor: Venkatramanan S, National College Autonomous, INDIA

Received: July 12, 2025

Accepted: October 8, 2025

Published: October 29, 2025

Copyright: © 2025 Zhang et al. This is an open access article distributed under the terms of the Creative Commons Attribution License, which permits unrestricted use, distribution, and reproduction in any medium, provided the original author and source are credited.

Data availability statement: All relevant data are within the manuscript.

Funding: This work was supported by the Doctoral Startup Fund of Yili Normal University (2025RCYJ06), the National Natural Science Foundation of China (41772130), and the Graduate Student Innovation Program of China University of Mining and Technology

Abstract

The Late Ordovician and Early Silurian transition is an important period of geological evolution, attracting increasing attentions. However, the cause for the biotic recovery from the end-Ordovician mass extinction has remained controversial. A set of black shales deposited in the Longmaxi Formation of the Early Silurian recorded the characteristics of the climate evolution after this extinction event, which played a crucial role in the biological recovery. In this paper, the shale of the Longmaxi Formation(LMX Fm.) in Well Yucan-6, Sichuan Basin is selected to analyze total organic carbon (TOC) and clay mineral composition, so as to address the climate evolution and its implications for the biotic recovery. The results show that the TOC content in the shale is higher at the bottom of the Longmaxi Formation in Well Yucan-6, and gradually decreases upward; the clay minerals are dominated by chlorite, illite, illite/smectite and chlorite/smectite mixed layer minerals, with no kaolinite and montmorillonite minerals being found. The clay minerals are mainly composed of illite/smectite mixed layer mineral with subordinate minerals of illite and chlorite, and a mall amount of chlorite/smectite mixed layer. Based on the characteristics of the illite and chlorite contents, and the ratios of illite/chlorite (I/C) and (smectite illit/smectite mixed layer mineral)/(illite+chlorite), i.e., (S+I/S)/(I+C), the paleoclimate evolution process of the Longmaxi Age is divided into three stages. Stage 1 is a climate evolution period of dry-wet, cold-hot rhythms; Stage 2 is a warm and humid climate evolution period, without obvious change in TOC content; and Stage 3 is a dry and cold climate evolution period, without obvious change in TOC. When combining these study results with the characteristics of the inorganic carbon isotope change trend in Silurian Epoch in the Tibet area and the whole world, the following conclusion has been drawn: The Early Silurian Longmaxi Age in Sichuan Basin was generally in a dry and cold environment, consistent with the characteristics of global paleoclimate evolution. These further indicates that the biotic recovery was

(2022WLKXJ002) and the Graduate Student Research Practice Innovation Program of Jiangsu Province (KYCX22_2600).

Competing interests: The authors have declared that no competing interests exist.

delayed by the dry and cold climatic conditions in the earliest Silurian following the Hirnantian glaciation. Until the relative warm and cold climatic conditions in the early Silurian, biotic recovery started with the ameliorative environments.

1. Introduction

The Late Ordovician - Early Silurian is a special period in geological history with frequently occurred anomalies on the surface. A series of major geological events, such as the convergence of extremely low glaciers, the frequent rises and falls of global sea level, the active inter-plate tectonic movement and the local volcanic eruption, occurred during this geological period, leading to revolutionary changes in the marine, continental and atmospheric environment. Among which, the first biological extinction event since the Phaneozoic happened in the Hernant Ice Age at end of the Ordovician Period, causing the extinction of 85% biological species on the Earth, next only to the Permian - Triassic extinction event on scale [1–5]. Research on the biological extinction event has been conducted from different perspectives, and opinions mainly focus on the following aspects: Global cooling during the ice age [6], marine hypoxia [7,8], volcanic activities [9–12], special paleogeographic location [13], and even the evolution of land plants [14,15] may cause the biological extinction. Some other studies show that this extinction event is a pulse-type event composed of two episidic sub-events [16] that are believed to happen one after another inferred from the characteristics of the high-resolution biodiversity curves [1,11,17]. The Longmaxi Formation is a set of black shales deposited after this extinction, which recorded the climate evolution characteristics after this event. Research on this formation can explain whether another extinction sub-event continuously happened afterward. Therefore, the Early Silurian Longmaxi Formation shale in the Sichuan Basin is selected as the object of this study. By using the characteristics and the ratios of the clay minerals in the shale as indicators, the climate evolution after the biological extinction event has been explored, and the continuity of this extinction event has been revealed through global comparison studies.

2. Geological setting

The metamorphic crystalline basement was formed by Xuefeng movement in Sichuan Basin [18]. The Sinian-Early Ordovician strata have evolved into a passive continental margin, and the Guangxi movement starting from the Late Ordovician controlled the formation of the Chuanzhong Uplift in the north, Qianzhong Uplift in the south, and Xuefeng Uplift in the southeast of the study area (Fig 1a) [19,20]. Due to the transformation of the tectonic cycle and superposition in Yanshanian Period, the folds in the study area occurred mainly in NE-NNE direction, and 3 sets of faults developed in NE, near SN and near EW direction respectively. The outcrop strata in this area mainly include the Cambrian, Ordovician, Silurian, Carboniferous, Permian, Triassic, Jurassic and Quaternary Systems. The study area is located on the southeastern margin of Sichuan Basin (Fig 1b), with strong tectonic and fault activities, large sedimentary thickness and serious erosion [20,21]. From northwest to southeast, the

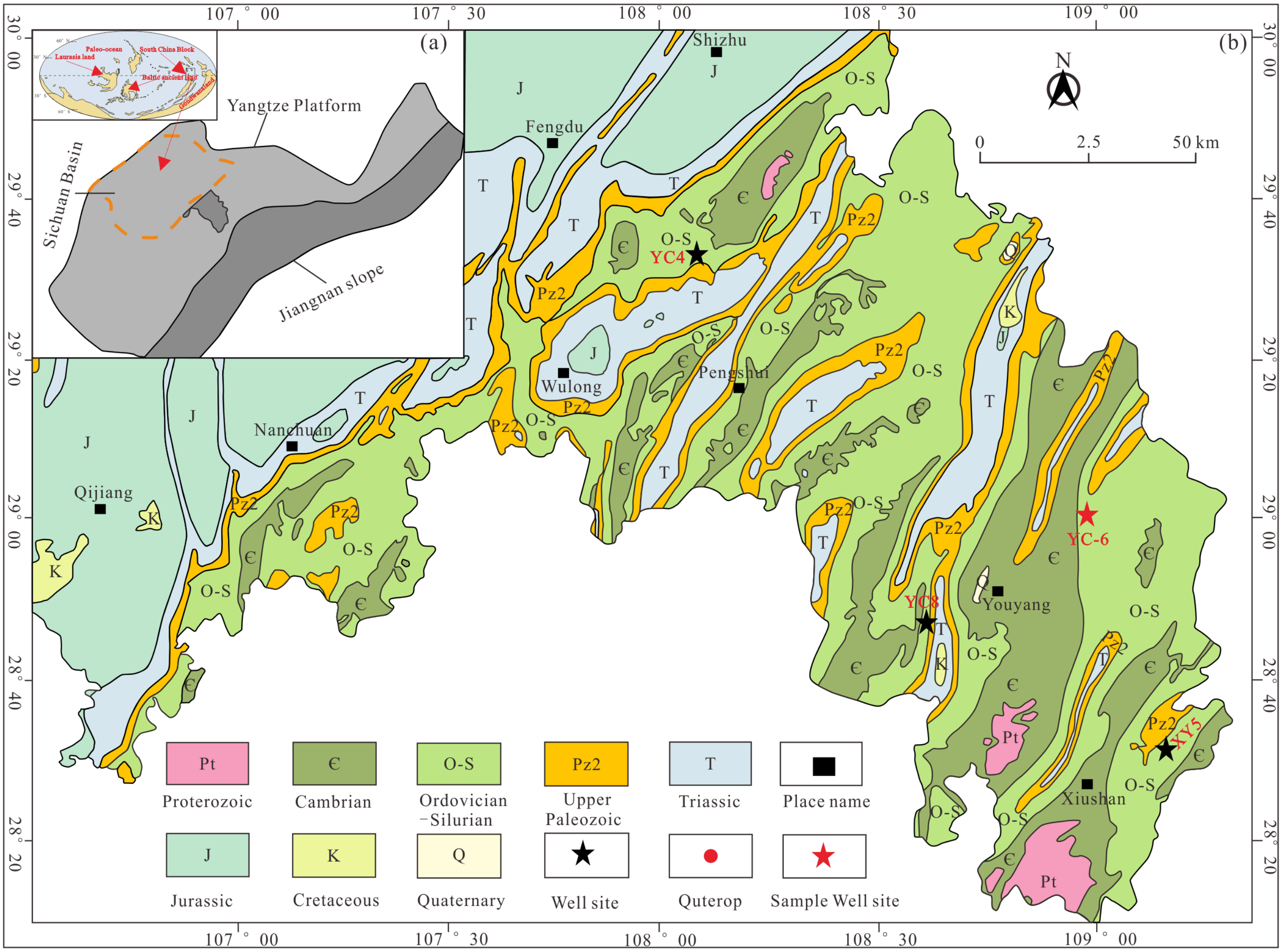

Fig 1. Regional geological background [19].

https://doi.org/10.1371/journal.pone.0335236.g001

tectonic pattern varies from trough fold to zonal fold in the southeast Chongqing [19]. The extensive shallow marine sediments developed due to tectonic uplifting, causing the Early-Middle Ordovician in a low-energy and anoxic environment [22]. Two global transgression events happened during the Late Ordovician-Early Silurian period and affected the Yangtze Platform and also had an important impact on the sedimentation of the Wufeng and Longmaxi Formations [23]. The study horizon mainly developed black carbonaceous shale and black siliceous rock of the Upper Ordovician Wufeng Formation with rich graptolite fossils, and black carbonaceous shale, light black argillaceous shale and light gray silty shale of the Lower Silurian Longmaxi Formation [24–26]. Up to now, the paleo biostratigraphic zone of the Wufeng-Longmaxi Formation has been classified and compared with the global biostratigraphic zone in detail [27–28].

3. Petrologic feature

The Longmaxi Formation in Well Yucan-6 is dominantly composed of carbonaceous shale, siliceous shale, silty shale and siltstone in lithology, with pyrite developing along the beddings and visible graptolite fossils. In the upper part of this

formation, the ripple laminations can be seen in the silty shale (Fig 2a) with rich organic matter in the cracks that can be observed under the microscope (Fig 2e-f). The fractures are relatively developed with carbonaceous siltstone and are filled with calcite on the fracture plane (Fig 2b) that can also be observed under the microscope (Fig 2g). In the lower part of this formation, a large amount of graptolite fossils have been discovered on the cross section of the carbonaceous shale (Fig 2c), with pyrite relatively developed and filled in disseminated form (Fig 2d). All these findings indicate that the Longmaxi Formation has experienced an evolution process with sea level changing from rise to fall, causing the development of pyrite and the relatively high content of carbon in the lower part of the formation, reflecting that the lower part of the formation was developed in a deep water and relatively anoxic reduction environment, which is conducive to the enrichment of organic matter.

a-Laminated shale; b-Shale with fissures filled by calcite; c-Shale with rich graptolite fossils; d-Shale developed with banded pyrite; e and f-microscopic characteristics of shale laminae under microscopes; g-Calcite veins of carbonaceous shale under microscopes

4. Analytical method

Well Yucan-6 is located in Youyang area, Chongqing suburbs, which is an exploration well deployed by Chongqing Institute of Geology and Mineral Resources. In this study, the drilling cores from Well Yucan-6 are taken as study object, and

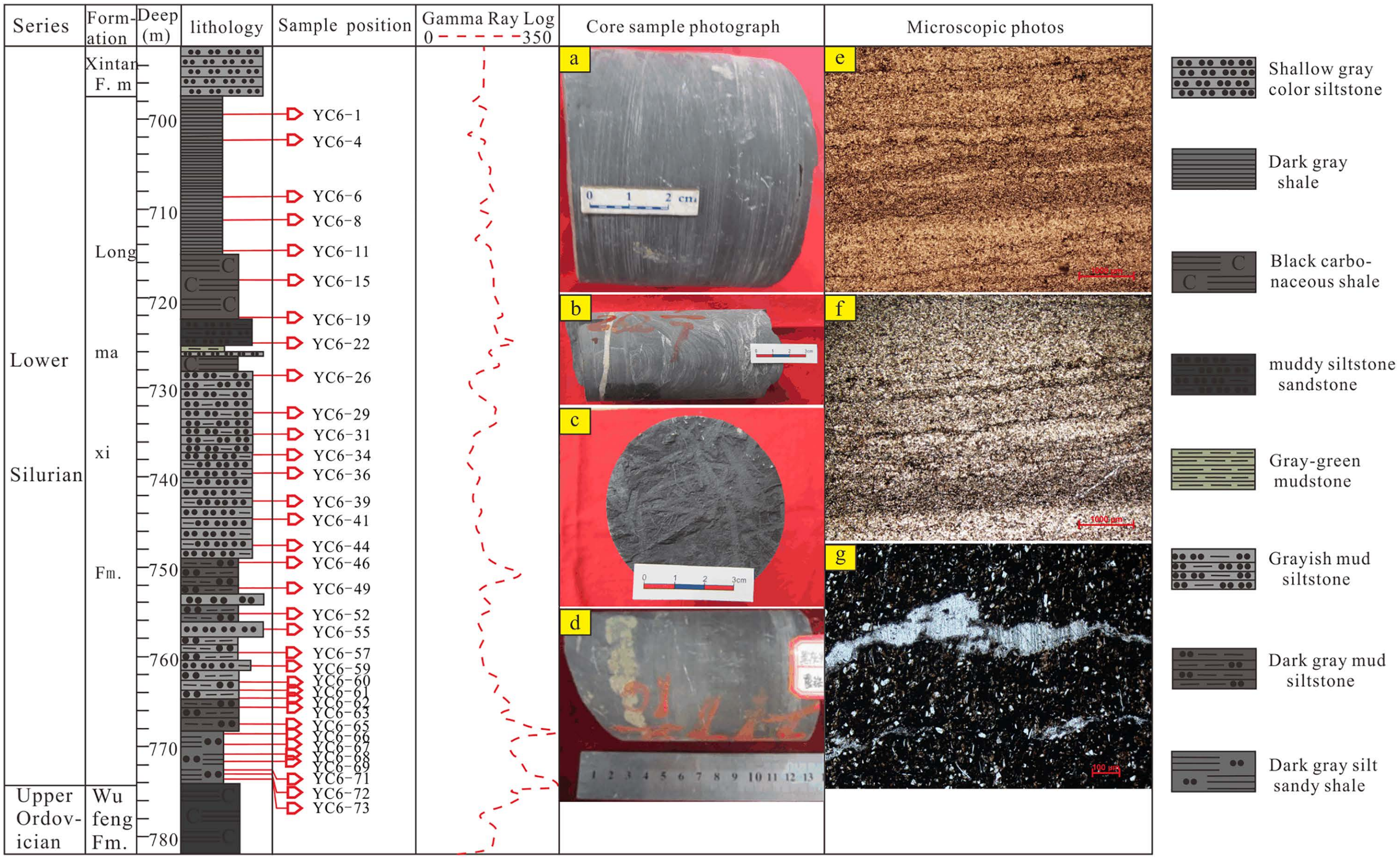

Fig 2. Lithologic column of Well Yucan-6 and sampling location.

https://doi.org/10.1371/journal.pone.0335236.g002

34 core samples have been collected considering the rock combination types of the Longmaxi Formation encountered in this well (Fig 2). The samples were pretreated in the Laboratory of China University of Mining and Technology. They were first crushed by using an electromagnetic crusher (DF-4) to 200 meshes, then packed into pollution-free and clean self-sealable bags after screening, and finally sent to the Chongqing Mineral Resources Supervision and Testing Center for TOC and clay mineral measurement. In addition, the core samples were also sent to the Laboratory of Hebei Institute of Geological Surveying and Mapping in original massive state for slice making, and then the thin slice identification studies were carried out in the Key Laboratory of Coalbed Methane and Accumulation Process of the Ministry of Education, China University of Mining and Technology.

4.1. Whole rock TOC measurement

The 100 mg 200 powder sample was weighed and put into a centrifuge tube. Then 4N HCl solution was added for 24 hours digestion by shaking. After that, the tube was put into a centrifuge for centrifugal treatment; next the supernatant was poured out and added with the deionized water, and treated with centrifugation again. After being treated with centrifugation in this way for 3 times, and the pH value of the supernatant finally became neutral when testing with pH paper. The treated supernatant was put into a freeze dryer to dry for 48 hours. The 30mg dried sample was weighed and wrapped in tin foil, and then put into MAT-253 elemental analyzer-isotope ratio mass spectrometer for measurement. The analysis error was less than 0.2‰, as shown in Table 1.

4.2. Whole rock clay mineral measurement

The type and relative content of clay minerals are measured by X-ray diffraction (XRD) analysis. Samples were first crushed to particles with the diameter less than 0.2 nm, and then soaked in distilled water and kept for more than 48 hours. Next, the clay minerals obtained from the suspension liquids were prepared into naturally directional slices (N slices), ethylene glycol saturated slices (EG slices) and heat-treated high-temperature slices (T slices) after heating treatment (550°/2h) for experimental analysis. The measurement was carried out by Chengdu Nanda Microstructure Quality Inspection Technical Service Co., LTD. The measuring instrument used was Dutch Panaco X'pert diffractometer with the following working conditions: Cu target radiation, 40kV operating voltage for X-ray tube, 40mA current, RS = 5.5 mm, 3° ~ 30° scanning angle (2θ) range, and 10°/min scanning speed. Since different types of clay minerals have different layer morphologies and contain interlayer materials, the basal spacing (d) and the base plane diffraction intensity of the clay minerals are different from each other. For example, the d (001) values of chlorite group and illite group materials are 1.41-1.435nm and 0.995-1.00nm, respectively, and the d (001) value of montmorillonite group materials varies greatly (1.2-1.6nm) and can be expand to 1.7nm only under the treatment of ethylene glycol. Therefore, the types of clay minerals can be identified based on the differences of their base spacing (d (001)) and base diffraction intensity [29]. The relative content of clay minerals is calculated by multiplying the characteristic diffraction peak intensity of each clay mineral in the sample by their respective weight coefficients, and set this value as 100%. In this way, the content percentage of each clay mineral can be calculated as commonly used weight coefficient [30,31]. The standard of the weight peak intensity used presently is as follows: The value of the 1.7nm diffraction peak intensity obtained after the treatment of ethylene glycol multiplied by 1 is taken as the weight peak intensity of montmorillonite; the 1.0nm diffraction peak intensity multiplied by 4 is taken as the weight peak intensity of illite; and the 0.7 nm diffraction peak intensity multiplied by 2 is taken as the weight peak intensity of kaolinite+chlorite. The content ratio of kaolinite and chlorite can be calculated directly from the ratio of the diffraction peak intensity of 0.356 ~ 0.358nm (kaolinite) and the diffraction peak intensity of 0.353nm (chlorite) [30,31]. The crystallinity of illite (evaluated by Kubler index) is expressed by the full wave at half maximum (fwhm) of the diffraction peak of illite d (001) measured by Jade software [30–33]. The calculation results are shown in Table 1.

Table 1. Clay mineral content and ratios in Yushen-6 well of Longmaxi Formation (LMX Fm.) in the study area.

Sample Number	Deep (m)	strata	TOC (%)	C	I	I/S	C/S	I/S*	C/S*	KI(°)	I/C	(S+I/S)/ (I+C)
YC6−1	697.3	LMX F. m	0.2	16	19	56	9	6	10	0.33	1.19	1.60
YC6−4	702.37	LMX F. m	0.6	11	37	46	6	7	12	0.42	3.36	0.96
YC6−6	708.71	LMX F. m	1.0	13	33	49	5	9	14	0.45	2.54	1.07
YC6–8	711.59	LMX F. m	1.0	10	21	62	7	9	12	0.47	2.10	2.00
YC6–11	714.6	LMX F. m	0.9	9	30	57	4	7	13	0.32	3.33	1.46
YC6–15	718.1	LMX F. m	1.3	10	24	58	8	9	11	0.36	2.40	1.71
YC6–19	722.53	LMX F. m	1.2	9	28	59	4	7	14	0.51	3.11	1.59
YC6–22	725.7	LMX F. m	0.9	9	26	60	5	7	12	0.53	2.89	1.71
YC6–26	729.4	LMX F. m	0.4	6	35	56	3	8	15	0.55	5.83	1.37
YC6–29	732.6	LMX F. m	0.4	6	29	62	3	9	13	0.47	4.83	1.77
YC6–31	734.9	LMX F. m	0.7	6	22	68	4	9	12	0.62	3.67	2.43
YC6–34	737.5	LMX F. m	0.5	9	28	61	2	8	16	0.37	3.11	1.65
YC6–36	739.8	LMX F. m	0.4	10	20	63	7	6	13	0.39	2.00	2.10
YC6–39	742.8	LMX F. m	0.6	9	24	63	4	5	12	0.41	2.67	1.91
YC6–41	744.50	LMX F. m	1.1	7	23	65	5	7	12	0.43	3.29	2.17
YC6–44	747.80	LMX F. m	0.9	8	29	59	4	7	12	0.53	3.63	1.59
YC6–46	749.40	LMX F. m	1.0	8	16	69	7	9	11	0.51	2.00	2.88
YC6–49	752.10	LMX F. m	0.4	8	15	73	4	8	13	0.56	1.88	3.17
YC6–52	754.90	LMX F. m	0.4	11	15	64	10	11	10	0.62	1.36	2.46
YC6–55	757.16	LMX F. m	0.4	8	17	71	4	8	13	0.31	2.13	2.84
YC6–57	759.57	LMX F. m	0.5	9	23	60	8	10	11	0.42	2.56	1.88
YC6–59	761.90	LMX F. m	0.4	6	34	57	3	8	12	0.64	5.67	1.43
YC6–60	762.9	LMX F. m	1.0	5	30	62	3	10	13	0.56	6.00	1.77
YC6–61	763.9	LMX F. m	1.0	5	15	76	4	8	14	0.58	3.00	3.80
YC6–62	764.1	LMX F. m	0.9	8	15	70	7	9	9	0.54	1.88	3.04
YC6–63	765.5	LMX F. m	1.2	6	14	76	4	8	13	0.62	2.33	3.80
YC6–65	767.9	LMX F. m	1.0	7	21	69	3	7	13	0.56	3.00	2.46
YC6–66	768.29	LMX F. m	1.1	7	16	72	5	8	12	0.52	2.29	3.13
YC6–67	769.5	LMX F. m	1.5	6	20	69	5	10	12	0.53	3.33	2.65
YC6–68	770.95	LMX F. m	2.0	9	11	72	8	7	10	0.54	1.22	3.60
YC6–69	771.4	LMX F. m	2.1	2	20	77	1	10	14	0.63	10.00	3.50
YC6–71	773.95	LMX F. m	3.1	5	13	77	5	9	12	0.54	2.60	4.28
YC6–72	774.1	LMX F. m	4.3	4	37	57	2	9	12	0.55	9.25	1.39
YC6–73	774.9	LMX F. m	4.8	8	46	40	6	7	10	0.57	5.75	0.74
average			1.2	7.94	23.71	63.38	4.97	8.12	12.26	0.50	3.42	2.23
minimum			0.2	2.00	11.00	40.00	1.00	5.00	9.00	0.31	1.19	0.74
maximum			4.8	16.00	46.00	77.00	10.00	11.00	16.00	0.64	10.00	4.28

Note: K: kaolinite C: chlorite; I: Illite; S: smectite; I/S: Illite/smectite interlayer mineral; C/S: chlorite/smectite interlayer mineral; I/S*: the amount of S (%) in Illite/smectite interlayer mineral. C/S*: the amount of S (%) in chlorite/smectite interlayer mineral.

https://doi.org/10.1371/journal.pone.0335236.t001

5. Results

The TOC content in the shale samples from the Longmaxi Formation in Well Yucan-6 ranges from 0.2% to 4.8%, with an average of 1.2%. Clay mineral chlorite (C) and illite contents range from 2.00% to 16.00% (avg. 7.94%) and from 11.00% to 46.00% (avg. 23.71%), respectively. The content of the illite/smectite (I/S) interlayer minerals is 40.00%-77.00%,

averaged 63.38%; the content the chlorite/smectite (C/S) mixed layer mineral is 1.00%-10.00%, averaged 4.97%; the content of smectite (S) in the illite/smectite (I/S) interlayer minerals is 5.00% -11.00%, averaged 8.12%; and the content of smectite (S) in the chlorite/smectite (C/S) interlayer minerals is 9.00%-16.00%, averaged 12.26% (Fig 3). The illite crystallinity (IC) is 0.31°-0.64°, averaged 0.50°; the ratio of illite/chlorite (I/C) is 1.19-10.00, averaged 3.42; the ratio of (kaolinite+illite/smectite interlayer minerals)/(illite+chlorite) [(S+I/S)/(I+C)] is 0.74-4.28, averaged 2.23. In general, the illite/smectite interlayer mineral has the highest content, followed by illite, and the least is the chlorite/smectite interlayer mineral (Table 1).

K: kaolinite C: chlorite; I: Illite; S: smectite; I/S: Illite/smectite interlayer mineral; C/S: chlorite/smectite interlayer mineral; I/S*: the amount of S (%) in Illite/smectite interlayer mineral. C/S*: the amount of S (%) in chlorite/smectite interlayer mineral.

6. Discussion

6.1. Reliability evaluation of clay mineral indicators

The clay mineral samples selected for measurement should be ensured not having been subjected to significant diagenesis, and the provenance changes should also be evaluated when the samples are used for paleoclimatic reconstruction. Only when the metamorphism degree of the clay minerals is not high and the provenance location has not changed greatly, the clay minerals can be used to reflect the paleoclimate evolution in the study area [32–33]. Illite crystallinity (IC) has been widely used as an indicator to infer the degree of metamorphism, with the IC values of 0.42° and 0.25° as threshold values of the following 3 metamorphism degree types: no metamorphism (>0.42°), approaching metamorphism (0.25°-0.42°), and slight metamorphism (< 0.25°). The IC values are calculated by using the Jade software to measure the full wave at half maximum (fwhm) in this study. The results show that the IC values of the illite of the Longmaxi Formation in Well Yucan-6 are all greater than 0.25°, indicating that the clay minerals were just approaching metamorphism or have not undergone any metamorphism. Therefore, the clay minerals can be used as an effective indicator in paleoclimate restoration [32–33] and reconstruction. Zhang et al. [20] used the Al_2O_3/TiO_2 ratio to explore the stability of the mineral source in the adjacent Well Youcan-2, and found that the distribution of the Al_2O_3/TiO_2 ratios in the shale from the Longmaxi Formation is relatively stable with only small fluctuations in the vertical direction, indicating that the mineral source Well Youcan-2 is consistent with that of Well Yucan-6. Therefore, the crystallinity angle of illite, combined with the provenance

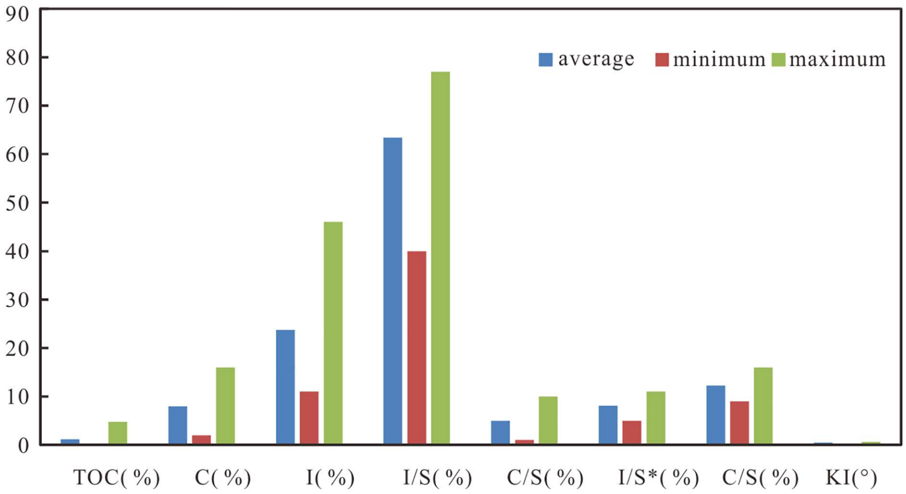

Fig 3. Histogram of TOC, clay minerals and illite crystallinity angle of Longmaxi Formation (LMX Fm.) in Well Yucan-6.

https://doi.org/10.1371/journal.pone.0335236.g003

characteristics of the adjacent Well Youcan-2, shows that the characteristics and the ratios of the clay minerals of the Longmaxi Formation in Well Yucan-6 can be used reliably in paleoclimate restoration, and thus can be used in the paleo-climate reconstruction of Longmaxi Age.

6.2. Paleoclimate evolution in the Early Silurian

6.2.1. *Clay mineral content and paleoclimate conditions.* Clay minerals are usually produced by weathering of the epigenic regolith, and thus can be used to indicate the weathering degree and paleoclimate characteristics. Different assemblage and content characteristics of the clay minerals at present are the eventual sedimentary response to the paleoclimate evolution. Kaolinite is formed by intense leaching of feldspar, pyroxene and mica [34–35] in a warm and humid climate. Chlorite is unstable under oxidation conditions [36] and can only be preserved in an environment where chemical weathering is inhibited [35], and is commonly believed to form under arid conditions [34]. Illite is formed in an environment with cold climate and little rain [32,37,38], and can be transformed into kaolinite under humid climate conditions [38,39]. Therefore, the relative increase in chlorite and illite contents indicates a dry and cold climate environment. The Late Ordovician-Early Silurian Epoch in Sichuan Basin experienced the first extinction event since the Phanerozoic Eon, and the evolution of the climate ever since was very complex. Fortunately, the clay minerals in the Longmaxi Fomation shale in Well Yucan-6 carried the abundant information on climate restoration after this event, thus can reflect the paleo-climate evolution characteristics. Based on the vertical variation characteristics of the contents and ratios of the clay minerals in Well Yucan-6, the Longmaxi Formation was divided into 3 sections (Fig 4). In Section 1, the organic carbon content shows a changing trend from higher values to lower ones; the chlorite content experiences a high - low - high change process, in which the changes happen quite frequently; the illite content experiences a high - low - high change process, similar to the change characteristics of the chlorite content (Fig 4). The IC values are all greater than 0.5, with no kaolinite and montmorillonite detected. All these indicate the Section 1 of the Longmaxi Formation was mainly in a climate that alternated frequently between warmth and cold, generally inherited the cold climate environment of the Hernant Ice Age [40]. In Section 2, chlorite values experience a high - low - high change process, in which the changes were relatively gentle; illite values also experiences a high - low - high change process, in which the changes happen not so frequently and more stably compared with Section 1. These indicate that Section 2 was in a climate that changed from relative warmth to cold and dryness, but generally being warm and humid. In Section 3, the change of chlorite content is relatively gentle on the whole, and illite shows the same change characteristics, with no montmorillonite and kaolinite detected, indicating that Section 3 of the Longmaxi Formation was still in a relatively cold and arid climate.

6.2.2. *I/C and (S+I/S)/(I+C) ratios and paleoclimate evolution.* In addition to the types of clay minerals that can be used to indicate the different climate environments, the content ratios of some clay minerals (such as montmorillonite/illite ratio, clay mineral/quartz ratio, illite chemical index) can also be used as weathering indicators to measure the degree of chemical weathering, with the greater values indicating the stronger degrees of chemical weathering [41]. Furthermore, diagenesis is also an important factor influencing the composition of clay minerals, whose metamorphism degree and effectiveness in paleoclimate restoration can be measured by the IC value [37]. It has been discussed in Section 5.1 that the IC values in the study area are all greater than 0.25° with most being greater than 0.42°, indicating that the clay mineral indicators are reliable and effective. In the early stage of the weathering process, chlorite is easily decomposed into clay minerals such as vermiculite, montmorillonite, and vermiculite/chlorite mixed layer mineral, while illite is easily remained in the rocks due to its high anti-weathering ability. Moreover, the weathering rate of chlorite is greater than that of illite. Therefore, the ratios of illite/chlorite (I/C) and (montmorillonite+illite/montmorillite mixed layer mineral)/ (illite+chlorite) [(S+I/S)/(I+C)] can be used to identify the weathering intensity and pedogenic environment of the clay minerals. The greater the I/C and (S+I/S)/(I+C) ratios are, the warmer and humid climate was; otherwise, the less these ratios are, the colder and drier climate was [32,42]. The I/C and (S+I/S)/(I+C) ratios of the clay minerals in Well Yucan-6 generally show an increasing – decreasing - rising

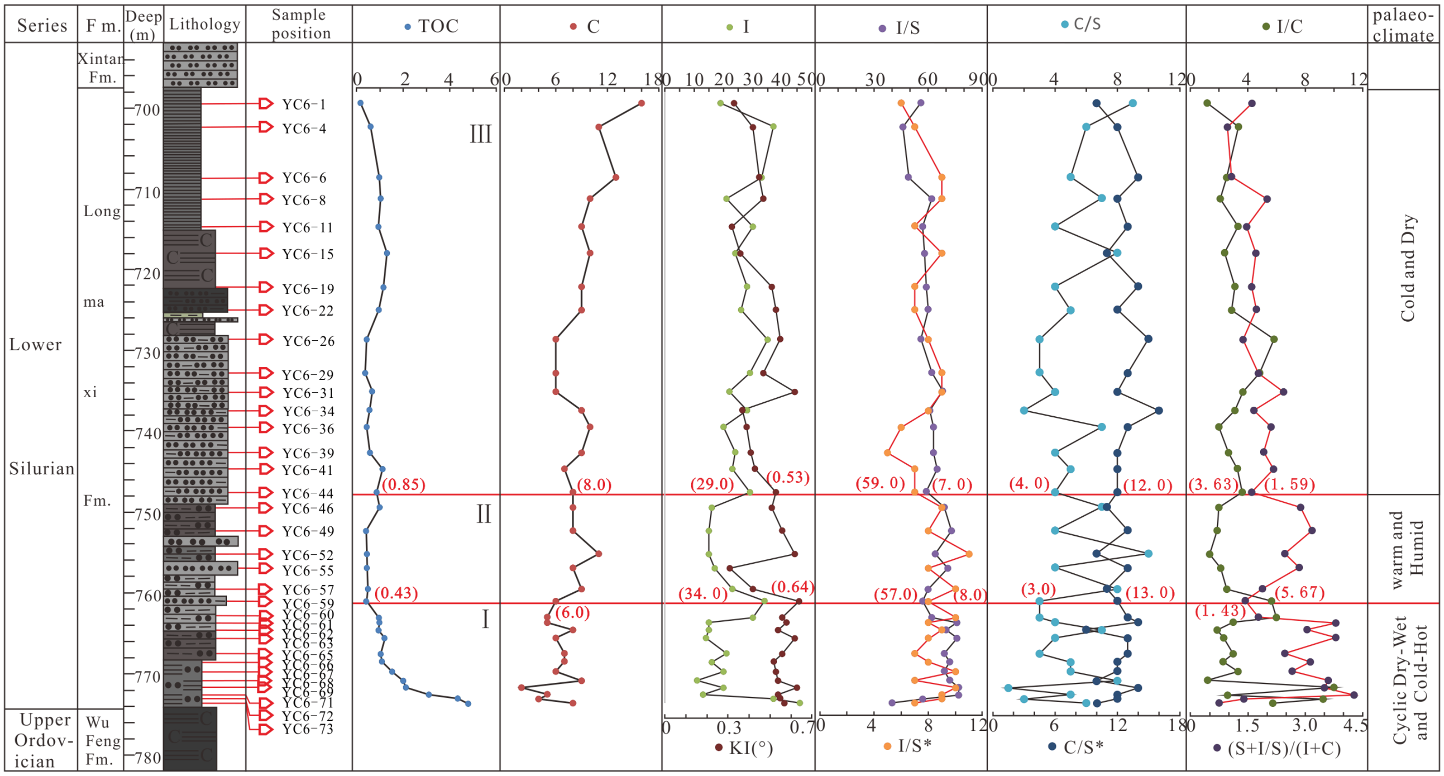

Fig 4. Content and ratio characteristics of clay minerals of Longmaxi Formation in Well Yucan-6 and paleoclimate evolution.

https://doi.org/10.1371/journal.pone.0335236.g004

change trend with frequent fluctuations. The I/C and (S+I/S)/(I+C) ratios of the clay minerals in the Section 1 of the Longmaxi Formation show an increasing - stably high - decreasing change trend with relatively frequent fluctuations (Fig 4), indicating that this section was in a climate environment where heat and cold alternated, similar to the climate environment characteristics inferred from illite and chlorite contents. The I/C and (S+I/S)/(I+C) ratios of the clay minerals in Section 2 of the Longmaxi Formation show a general increasing – decreasing - increasing change trend with more frequent fluctuations, indicating that this section was basically in a warm and humid climate environment when combining with the change characteristics of chlorite and illite contents (Figure 4). The I/C and (S+I/S)/(I+C) ratios of the clay minerals in Section 3 of the Longmaxi Formation generally show an increasing trend in ratio values with less fluctuations. Combined with the change characteristics of chlorite and illite contents, the change trend of these ratios indicates that Section 3 was generally in a cold and arid climate environment. The studies of the climate evolution and the characteristics of the TOC content changes of the 3 Sections of the Longmaxi Formation show that the climate environment with frequent alternation of heat and cold is not only the main cause of the biological extinction event, but also the main reason for the enrichment of organic matter.

In summary, the content and ratio characteristics of the clay minerals in the Longmaxi Formation show that the climate evolution in Longmaxi Age can be generally divided into 3 stages. Among which, Stage 1 mainly shows an alternating heat and cold climate environment; Stage 2 is dominated by a warm and humid environment, and Stage 3 is dominated by a cold and arid environment. Kaolinite and montmorillonite minerals have not been found in the Longmaxi Formation. Generally, this formation was in a cold and arid environment, the 3 stages being warm, humid, cold or arid as divided in this study are just in a relative perspective.

Note: K: kaolinite C: chlorite; I: Illite; S: smectite; I/S: Illite/smectite interlayer mineral; C/S: chlorite/smectite interlayer mineral; I/S*: the amount of S (%) in Illite/smectite interlayer mineral. C/S*: the amount of S (%) in chlorite/smectite interlayer mineral

6.3. *Global comparison of paleoclimate evolution in the Early Silurian*

The Early Silurian Period has been considered as a stable greenhouse period, with little or no glacier cover on both south and north poles, and the control of latitude over climate was weak [40]. However, other studies on large amounts of geochemical indicators revealed that the Early Silurian Period was in a unstable climate environment, and the existence of the Early Silurian glaciers had also been proved. Up to now, no consensus on the climate evolution of the Early Silurian Period has been reached [40,43]. In this study, the paleoclimate evolution is divided into 3 stages based on the indicative roles of the chlorite and illite content and characteristic indexes of the clay minerals in the Longmaxi Formation in Well Yucan-6, Southeastern Sichuan Basin. Stage 1 was dominated by a dry, wet, cold and heat alternating environment. When compared with the distribution of the chemical index of alteration (CIA corr) in Longmaxi Formation in Well Budi-1, Sichuan Basin reported by Ran et al. [44], it can be seen the CIA corr values are low and fluctuate in this stage, indicating a moderate weathering degree (Fig 5). The inorganic carbon isotope values in Xainza region of Tibet in Stage 1 show a high – low – high change process, similar to the global inorganic carbon isotope evolution curves [45]. The increase of inorganic carbon isotope values indicates the increase of terrigenous clastic input in the source area, and similarly the decrease of inorganic carbon isotope values indicates the decrease of terrigenous clastic input in the source

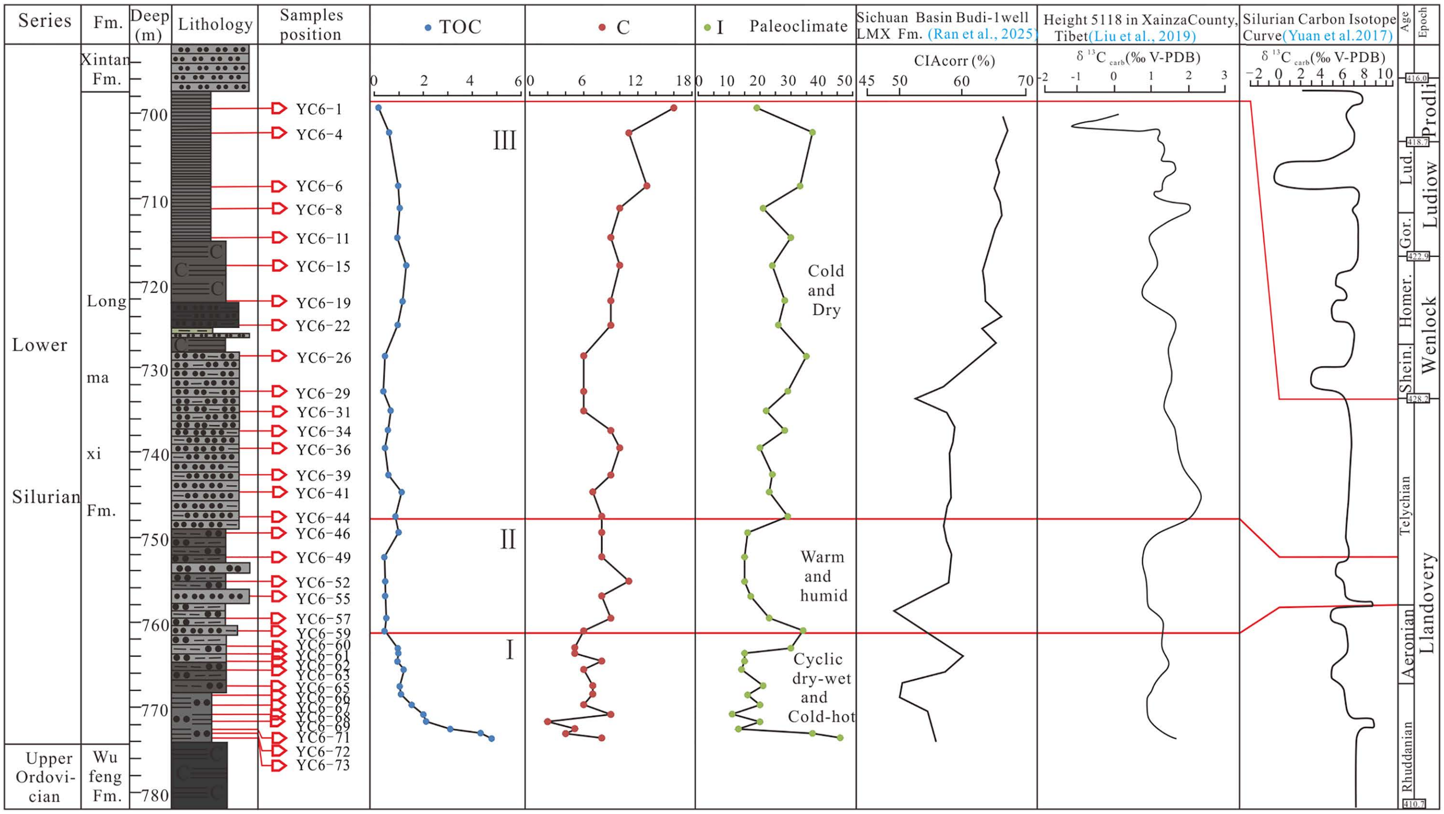

Fig 5. Comparison of climate change in Silurian Longmaxi Formation in the study area and carbon isotope curve in global Landovian Series.

https://doi.org/10.1371/journal.pone.0335236.g005

area. In general, the increase and decrease of inorganic carbon isotope values reflect the increase and decrease of chemical weathering degree respectively, and accordingly reflect the warm and humid climate and the wet and dry climate respectively. These results are consistent with the climate characteristics inferred from the clay mineral indicators of Well Yucan-6, indicating that the Early Silurian Longmaxi Stage (Stage 1) was mainly in a dryness, wetness, cold and heat alternating climate, and has global characteristics. Stage 2 shows a warm and humid climate, basically consistent with the climate characteristics indicated by the CIA corr of the shale of Longmaxi Formation in Well Budi-1, Sichuan Basin, as described by Ran et al. [44]. Although the CIA corr values in Stage 2 are lower than those in Stage 1, they are generally range between 60−70, indicating a moderate weathering degree, consistent with the result derived from inorganic carbon isotope in Xainza region of Tibet [43], and the result indicated by illite content curve characteristics in particular, and also the global inorganic carbon isotope curve [45]. All these indicate that Stage 2 of Longmaxi Age of the Early Silurian has returned to a warm and humid climate environment, which is consistent with the climate evolution characteristics in Tibet and the world. Stage 3 was dominated by a dry and cold climate, with the content values of chlorite and illite increasing significantly and less fluctuations compared with Stage 2, similar to the climate characteristics indicated by the increase and decrease of the CIA corr values of Longmaxi shale in Well Budi-1, Sichuan Basin [44], the carbon isotope values of inorganic carbon in Tibet [43], the illite content curve in particular, and also the global inorganic carbon isotope curve [45]. All these indicate that the climate environment in Stage 3 of Longmaxi Age of the Early Silurian has changed from a warm and humid climate environment in the middle Longmaxi Age to a dry and cold climate environment in the late Longmaxi Age, in sync with the climate evolution of Tibet and the world.

Note: K: kaolinite; C: chlorite; I: Illite; S: smectite; I/S: illite/smectite interlayer mineral; C/S: chlorite/smectite interlayer mineral; I/S*: the amount of S (%) in illite/smectite interlayer mineral; C/S*: the amount of S (%) in chlorite/smectite interlayer mineral; CIAcorr: Chemical Index of Alteration corrected; δ^{13}Ccarb(V-PDB): Inorganic carbon isotope

Through the discussion on the climate evolution characteristics of the Early Silurian Longmaxi Age in the study area and the whole Sichuan Basin, and the comparation studies with the evolution characteristics of the inorganic carbon values in Tibet and the whole world, it is found that the climate of the Early Silurian Langdovian Series in Sichuan Basin, derived from the climate of the global Early Silurian Langdovian Series, has obviously experienced the dryness, wetness, cold and heat alterations, and has not returned directly to the warm and humid environment of the Upper Ordovician Kaidian Stage. This may be influenced by the volcanic eruptions and the silicate weathering in the source area. The density of the volcanic ash in the shale of Wufeng Formation in Sichuan Basin is 6.00 layers/1 cm with an average thickness of 1 cm, much thicker than that of the volcanic ash layer of Longmaxi Formation with the volcanic ash density of 2.40 layers/1 cm and an average thickness of 0.34 cm (the density of the upper part being 0.27 layers/1 cm and an average thickness being 0.35 cm) [46]. These indicate that the volcanic eruptions were more frequent in the Wufeng Age and less frequent in the Longmaxi Age. The volcano eruption can release a large amount of CO_2 into the atmosphere, causing an obvious greenhouse effect and an increase in temperature, and accordingly resulting in a warmer and more humid climate. However, with the emergence of the global Hirnantian Ice Age [40,47], the temperature dropped sharply, with the Gondwana Glacier extending to South China [47–53]. As the Hernant Ice Age ended, the volcano eruption frequency was much less in the Early Silurian Langdovian Series (Longmaxi Age) although volcanoes still erupted [54]. Accordingly, the amount of CO_2 emission flux to the atmosphere caused by the volcano eruptions decreased, and the climate did not directly return to the humid and warm one as in the Wufeng Age, and was generally in a dry and cold climate environment, with intermittent temperature rising process. In this study, no montmorillonite and kaolinite have been found in the clay minerals of the Longmaxi Formation shale in Well Yucan-6, also verifying that the Sichuan Basin still inherited the climate environment of the Hernant Ice Age in the early Silurian, which is consistent with the previous study results [50–55]. It is believed that the glaciers still existed in the Early Silurian, indicating that there may still be small-scale biological extinction events in the Longmaxi Age, and the biological recovery would probably begin after the end of the Early Longmaxi Age.

7. Conclusion

(1) The paleoclimate evolution process of the early Silurian Longmaxi period in Sichuan Basin has been divided into 3 stages based on the content and characteristic ratio of the clay minerals. Among which, Stage 1 is in the early Longmaxi period, dominated by alternating cold, hot, dry and wet climates; Stage 2 is in the middle Longmaxi period, with the climate being mainly warm and humid; Stage 3 is in the late Longmaxi period, with the climate being mainly cold and arid. As a whole, it is believed that the Longmaxi period is still in a quite cold climate, with only relatively frequent temperature fluctuations.

(2) The Early Silurian Longmaxi period in Sichuan Basin has been revealed in a cold and arid climate environment by the similaritis between the climate evolution characteristics of this period in Sichuan Basin and the inorganic carbon isotope characteristics of this period in Tibet and in the world. Such similaritis indicate that the cold and arid climate environment in the Early Silurian Longmaxi period is globally developed, rather than occurring in Sichuan Basin only. It further suggests that the Early Silurian inherited the cold climate characteristics of the Late Ordovician Hernant Ice Age, and therefore the biotic recovery would probably began after the end of the Longmaxi period and ever since gradually returned to the biological property before the extinction event.

Author contributions

Conceptualization: Zhibo Zhang.

Data curation: Zhibo Zhang.

Formal analysis: Zhibo Zhang, Huan Li.

Funding acquisition: Zhibo Zhang, Yinghai Guo.

Investigation: Zhibo Zhang, Jiaming Zhang, Huan Li, Yinghai Guo.

Methodology: Jiaming Zhang, Hengye Wei.

Project administration: Yinghai Guo.

Resources: Hengye Wei, Yinghai Guo, Chunlin Zeng.

Software: Chunlin Zeng.

Supervision: Huan Li, Chunlin Zeng.

Writing – original draft: Chunlin Zeng.

Writing – review & editing: Chunlin Zeng.

References

1. Rong JY, Fan JX, Miller AI. Dynamic patterns of latest Proterozoic-Paleozoic-Early Mesozoic marine biodiversity in South China. Geol J. 2007;42(3/4):431–54.
2. Berner RA. Atmospheric carbon dioxide levels over phanerozoic time. Science. 1990;249(4975):1382–6. https://doi.org/10.1126/science.249.4975.1382 PMID: 17812165
3. Yapp CJ, Poths H. Ancient atmospheric C02 pressures inferred from natural goethites. Nature. 1992;355(6358):342–4. https://doi.org/10.1038/355342a0
4. Brenchley PJ, Marshall JD, Carden GAF, Robertson DBR, Long DGF, Meidla T, et al. Bathymetric and isotopic evidence for a short-lived Late Ordovician glaciation in a greenhouse period. Geology. 1994;22(4):295–8. https://doi.org/10.1130/0091-7613(1994)022<0295:baiefa>2.3.co;2
5. Finnegan S, Bergmann K, Eiler JM, Jones DS, Fike DA, Eisenman I, et al. The magnitude and duration of Late Ordovician-Early Silurian glaciation. Science. 2011;331(6019):903–6. https://doi.org/10.1126/science.1200803 PMID: 21273448
6. Stanley SM. Temperature and biotic crises in the marine realm. Geol. 1984;12(4):205. https://doi.org/10.1130/0091-7613(1984)12<205:tabcit>2.0.co;2

7. Bond DPG, Grasby SE. Late Ordovician mass extinction caused by volcanism, warming, and anoxia, not cooling and glaciation. Geology. 2020;48(8):777–81. https://doi.org/10.1130/g47377.1
8. Longman J, Mills BJW, Manners HR. Late Ordovician climate change and extinctions driven by elevated volcanic nutrient supply. Nat Geosci. 2021;14:924–9.
9. Histon K, Klein P, Schönlaub HP. Lower Palaeozoic K-bentonites from the Carnic Alps, Austria. Aust J Earth Sci. 2007;100(1):26–42.
10. Huff WD. Ordovician K-bentonites: Issues in interpreting and correlating ancient tephras. Quatern Int. 2008;178(1):276–87.
11. Hu Y, Sun W, Ding X. Volcanic event at the Ordovician-Silurian boundary: The message from K-bentonite of Yangtze Block. Acta Petrol Sinica. 2009;25(12):3298–308.
12. He L, Wang YP, Chen DF. Geochemical features of sedimentary environment and paleoclimate during Late Ordovician to Early Silurian in southern Sichuan Basin. Geochimica. 2021;50(06):623–34.
13. Saupe EE, Qiao H, Donnadieu Y. Extinction intensity during Ordovician and Cenozoic glaciations explained by cooling and palaeogeography. Nat Geosci. 2020;13:65–70.
14. Lenton TM, Crouch M, Johnson M. First plants cooled the Ordovician. Nat Geosci. 2012;5:86–9.
15. Shen J, Pearson A, Henkes GA. Improved efficiency of the biological pump as a trigger for the Late Ordovician glaciation. Nature Geoscience. 2018;11(6):510–4.
16. Harper DAT, Hammarlund EU, Rasmussen CMØ. End Ordovician extinctions: A coincidence of causes. Gondwana Research. 2014;25(4):1294–307. https://doi.org/10.1016/j.gr.2012.12.021
17. Wilde P, Berry WBN. Destabilization of the oceanic density structure and its significance to marine "extinction" events. Palaeogeography Palaeoclimatology Palaeoecology. 1984;48(2–4):143–62.
18. Ran B, Liu S, Jansa L, Sun W, Yang D, Ye Y, et al. Origin of the Upper Ordovician–lower Silurian cherts of the Yangtze block, South China, and their palaeogeographic significance. J Asian Earth Sciences. 2015;108:1–17. https://doi.org/10.1016/j.jseaes.2015.04.007
19. Luo Q, Zhong N, Dai N, Zhang W. Graptolite-derived organic matter in the Wufeng–Longmaxi Formations (Upper Ordovician–Lower Silurian) of southeastern Chongqing, China: Implications for gas shale evaluation. International J Coal Geol. 2016;153:87–98. https://doi.org/10.1016/j.coal.2015.11.014
20. Zhang Z, Guo Y, Zhao D, Zhang J, Zeng C, Li Y. Control Model of Organic Shale Enrichment by Terrigenous Weathering in Wufeng Formation–Longmaxi Formation, Southeast Sichuan, China. Minerals. 2023;13(6):761. https://doi.org/10.3390/min13060761
21. Hu H, Zhao D. Evaluation of the brittleness of deep shale reservoirs and optimization of the comprehensive brittleness index evaluation path-an example of the Wufeng and Longmaxi formations in the Z-3 well. Unconventional Oil and Gas. 2023;10(01):84–92.
22. LIU D, Wang M, Zhao T, et al. Quantitative characterization of nanopore structure classification in Wufeng Formation-Longmaxi Formation shale reservoirs-an example of WX-1 well in Wuxi area[J]. Unconventional Oil and Gas, 2023, 10 (01): 93–103.
23. Mu C, Zhou K, Liang W. Early Paleozoic sedimentary environment of hydrocarbon source rocks in the Middle-Upper Yangtze Region and petroleum and gas exploration. Acta Geol Sin. 2011;85:526–32.
24. Zhao D, Guo Y, Zhu Y. Review on the evaluation system of deep marine shale reservoirs for accurate evaluation and development. Unconventional Oil and Gas. 2022;9(02):1–7.
25. Yan D, Wang H, Fu Q, Chen Z, He J, Gao Z. Geochemical characteristics in the Longmaxi Formation (Early Silurian) of South China: Implications for organic matter accumulation. Marine Petroleum Geol. 2015;65:290–301. https://doi.org/10.1016/j.marpetgeo.2015.04.016
26. Liu T, Zhang Y, Zhao D. Stratigraphic delineation of Wufeng Formation-Longmaxi Formation shale in Jiaoshiba area, Sichuan Basin, and prediction of gas-bearing properties of the shale - taking JY-2 well as an example. Unconventional Oil and Gas. 2022;9(02):34–41.
27. Xu C, Jiayu R, Mitchell CE, Harper DAT, Junxuan F, Renbin Z, et al. Late Ordovician to earliest Silurian graptolite and brachiopod biozonation from the Yangtze region, South China, with a global correlation. Geol Mag. 2000;137(6):623–50. https://doi.org/10.1017/s0016756800004702
28. Jiang Z. Main controlling factors and depositional patterns of organic matter enrichment in the Wufeng Formation-Longmaxi Formation, Sichuan Basin. Nanchang: East China University of Technology. 2018.
29. Grim RE. Crystal Structures of Clay Minerals and Their X-Ray Identification. Earth-Science Reviews. 1982;18(1):84–5. https://doi.org/10.1016/0012-8252(82)90013-7
30. Johns WD, Grim RE, Brad WF. Quantitative Estimations of Clay Minerals by Diffraction Methods. SEPM JSR. 1954;Vol. 24. https://doi.org/10.1306/d42697b5-2b26-11d7-8648000102c1865d
31. Biscaye PE. Mineralogy and Sedimentation of Recent Deep-Sea Clay in the Atlantic Ocean and Adjacent Seas and Oceans. Geol Soc America Bull. 1965;76(7):803. https://doi.org/10.1130/0016-7606(1965)76[803:masord]2.0.co;2
32. Chen T, Wang H, Zhang ZQ. Clay minerals as indicators of paleoclimate. Acta Petrologica et Mineralogica. 2003;22(4):416–20.
33. Wang HJ, Zhou J. On the indices of illite crystallinity. Acta Petrologica Sinica. 1998;(03):128–38.
34. Zhang HY, Yang WT. Research status of deep-time paleoclimate analysis methods for terrestrial pulveryte and paleosols. Acta Sedimentologica Sinica. 2023;41(02):333–48.
35. Tang YJ, Jia JY, Xie XD. Environment significance of clay minerals. Earth Science Frontiers. 2002;9(2):337–44.

36. Bain DC. The weathering of chloritic minerals in some scottish soils. Journal of Soil Science. 1977;28(1):144–64. https://doi.org/10.1111/j.1365-2389.1977.tb02303.x
37. Sun QF, Colin C, Chen FH. A discussion on the factors affecting formation and quantity of clay minerals in climatic and environmental researches. Acta Petrologica et Mineralogica. 2011;30(2):291–300.
38. Zhang ZB, Guo YH, Wei HY. The intensification of prolonged cooling climate-exacerbated late Ordovician–early Silurian mass extinction: A case study from the Wufeng Formation–Longmaxi Formation in the Sichuan Basin. J Mar Sci Eng. 2023;11:1401.
39. Chamley H. Clay sedimentology. Berlin: Spring-Verlag. 1989.
40. Yang X, Yan D, Zhang L, et al. Current status of research on the paleoceanic environmental transition and its genesis mechanism during the Hernandez Ice Age. Acta Sedimentologica Sinica. 2018;36(02):319–32.
41. Gao Y, Gao Y, Ibarra DE, Du X, Dong T, Liu Z, et al. Clay mineralogical evidence for mid-latitude terrestrial climate change from the latest Cretaceous through the earliest Paleogene in the Songliao Basin, NE China. Cretaceous Research. 2021;124:104827. https://doi.org/10.1016/j.cretres.2021.104827
42. Song EP. Clay mineralogy of the gerze sediments and palaeoclimatic evolution in the central Qinghai-Tibetan Plateau [D]. Wuhan: China University of Geosciences. 2014.
43. Liu Y, Wu B, Gong Q, Cao H. Geochemical characteristics of the lower Silurian Longmaxi Formation on the Yangtze Platform, South China: Implications for depositional environment and accumulation of organic matters. Journal of Asian Earth Sciences. 2019;184:104003. https://doi.org/10.1016/j.jseaes.2019.104003
44. Ran B, Yang XR, Qi S. The evolution of continental weathering and upwelling in early Silurian and the implications for the organic matter accumulations in the shales of Longmxi formation. Bulletin of Geological Science and Technology. 2025. https://doi.org/10.19509/j.cnki.dzkq.tb20240678
45. Yuan T. Study on Ordovician-Silurian carbon isotope anomalies and paleomarine environment in Shenzha area, Tibet. Chengdu: Chengdu University of Technology. 2017.
46. Cramer BD, Brett CE, Melchin MJ, Männik P, Kleffner MA, Mclaughlin PI, et al. Revised correlation of Silurian Provincial Series of North America with global and regional chronostratigraphic units and δ 13 C carb chemostratigraphy. LET. 2011;44(2):185–202. https://doi.org/10.1111/j.1502-3931.2010.00234.x
47. Du X, Jia J, Zhao K. Characteristics of the development of deep-time volcanic ash layers in the Ordovician-Silurian transition in the Yangzi region and their influence on the deposition of organic-rich shales. Journal of Central South University (Natural Science Edition). 2022;53(09):3509–21.
48. Qiu Z, Lu B, Chen ZH. Discussion of the relationship between volcanic ash layers and organic enrichment of black shale: A case study of the Wufeng-Longmaxi gas shales in the Sichuan Basin. Acta Sedimentologica Sinica. 2019;37(6):1296–308.
49. Mu X, Ge X, Yu Q. Paleoclimate and material source characteristics of the black shale of the Wufeng-Longmaxi Formation in southwest Sichuan: geochemical records from the Xindi 2 well. J Paleogeography. 2019;21(05):835–54.
50. Ge X. Event deposition and organic matter enrichment during the Ordovician-Silurian succession in the eastern Sichuan Basin. Beijing: China University of Geosciences (Beijing). 2020.
51. Fischer AG. Long-term climatic oscillations recorded in stratigraphy. In: Berger WH, editor. Climate in Earth History. National Academy of Sciences. 1983. p. 97–104.
52. Page A, Zalasiewicz J, Williams M, et al. Were transgressive black shales anegative feedback modulating glacioeustasy in the Early Palaeozoic Icehouse? In: Williams M, Haywood A.M, Gregory F.J, Schmidt D.N. (Eds.). Deep-Time Perspectives on Climate Change: Marrying the Signal from Computer Models and Biological Proxies: Special Publication of the Geological Society of London. The Micropalaeontological Society. 2007, pp: 123-156.
53. Shen J-J, Yan J-K, Wang P-W, Wang Y-M, Zhou L, Ji Y-B, et al. Upwelling current and its relationship to the enrichment of organic matter in the Lower Silurian Longmaxi Formation, northern Chongqing–western Hubei area, southern China. J Palaeogeography. 2024;13(4):993–1015. https://doi.org/10.1016/j.jop.2024.08.005
54. Cai Q-S, Hu M-Y, Oumar IK, et al. Petrological and geochemical characteristics of the OrdovicianeSilurian black shale in eastern Sichuan and western Hubei, South China: Differential sedimentary responses to tectonism and glaciation. J Palaeogeography. 2023;12(1):129–52. https://doi.org/00291
55. Shi Z-S, Zhou T-Q, Song H-B, Niu Y-B, Sun S-S. Vertical variations in planar lamination of marine shale: Elucidating hydrodynamic changes during the Ordovician–Silurian transition on the Upper Yangtze Block. J Palaeogeography. 2025;14(1):221–44. https://doi.org/10.1016/j.jop.2024.09.003
